# The impact of Mediterranean diet adherence on allergic diseases: a systematic review and meta-analysis

**DOI:** 10.1017/jns.2026.10125

**Published:** 2026-07-20

**Authors:** Eleftheria Panagiotou, Eleni Andreou, Stella A. Nicolaou

**Affiliations:** Department of Life Sciences, School of Life and Health Sciences, University of Nicosia, Cyprus

**Keywords:** allergies, eczema, food allergies, Mediterranean diet, respiratory allergies

## Abstract

Allergies are a growing public health concern worldwide. This meta-analysis investigated the association between adherence to the Mediterranean diet (MD) and the occurrence/severity of respiratory allergic conditions. Electronic databases (PubMed, Scopus, Science Direct, and EBSCO) were searched for studies published up to April 2024. Eligible studies reported OR with 95% CI for asthma, wheezing (respiratory symptom associated with allergic airway disease), rhinitis, or eczema. Food allergy was evaluated only qualitatively due to insufficient data. The risk of bias was assessed using the National Heart, Lung, and Blood Institute tool, and random-effects models were applied to account for heterogeneity. A total of twenty-three studies involving 111,583 participants met the inclusion criteria. Individual studies could contribute to more than one condition-specific analysis if they reported outcomes for multiple allergic conditions. Pooled analyses demonstrated a significant protective effect of MD against asthma (*n* = 11, log OR: −0.18; 95% CI: −0.34, −0.02), wheezing (*n* = 6, log OR: −0.29; 95% CI: −0.56, −0.03), and rhinitis (log OR: −0.54; 95% CI: −1.00, −0.08), with stronger effects in Mediterranean populations. No significant association was observed with eczema (pooled log OR: 0.09; 95% CI: −0.02, 0.20). Heterogeneity was moderate across analyses. Publication bias was evaluated using forest plots and Egger’s test. These findings suggest that MD adherence was associated with lower odds of asthma, wheezing, and rhinitis, but does not influence eczema risk. Further longitudinal studies with standardised methodologies are needed to confirm causality. Promoting MD adherence may offer a valuable population-level strategy for allergy prevention. Systematic review registration: PROSPERO-CRD42023408724.

## Graphical abstract

**Figure:**
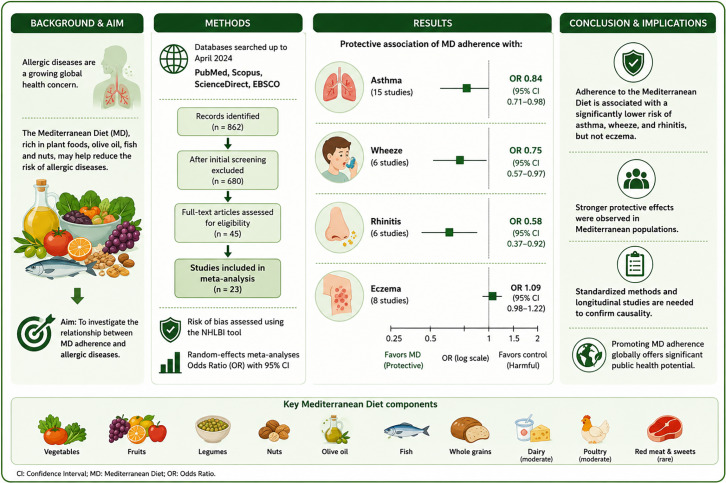

## Introduction

Allergies affect over 20% of the global population and are recognised by the WHO as one of the most prevalent chronic diseases.^(1–5)^ They pose a substantial economic and social burden through healthcare costs, productivity loss, and reduced quality of life.^(6)^ The most common allergies that people develop include eczema, asthma, allergic conjunctivitis, allergic rhinitis, and food allergies (FA).^(3,7)^ At the cellular level, allergic diseases (AD) arise from type 2 immune responses that engage both innate and adaptive immunity.^(8)^ The process often begins when environmental triggers (allergens) such as food, pollutants, or animal dander compromise epithelial barrier integrity in the skin, airways, or gut, prompting damaged epithelial cells to release alarmins (thymic stromal lymphopoietin, IL-25, and IL-33). These alarmins activate type 2 innate lymphoid cells (ILC2s) and dendritic cells (DCs), effectively linking innate defences to the adaptive immune response. During sensitisation, allergen-loaded DCs prime Th2 lymphocytes in draining lymph nodes. Once activated, Th2 cells produce IL-4, IL-5, and IL-13. IL-4 and IL-13 drive *B*-cell class switching towards allergen-specific antibodies, immunoglobulin E (IgE), while IL-5 recruits eosinophils that sustain chronic tissue inflammation.^(8)^ The IgE produced binds to FcϵRI receptors on mast cells and basophils; on subsequent allergen exposure, cross-linking of surface-bound IgE triggers degranulation and the release of histamine, tryptase, leukotrienes, PG, and pro-inflammatory cytokines. ILC2s further reinforce this inflammatory milieu through sustained IL-5 and IL-13 production, contributing to mucus hypersecretion and smooth-muscle contraction independently of antigen-specific immunity. Clinically, these cascades produce symptoms ranging from urticaria, rhinorrhoea, and bronchospasm to life-threatening anaphylaxis, and when type 2 inflammation persists, it can drive structural tissue changes such as subepithelial fibrosis and airway remodelling that underpin chronic conditions including asthma and atopic dermatitis.^(3,7,8)^ Diagnosis involves a detailed clinical history, physical examination, and tests, such as skin prick testing or measurement of total and/or allergen-specific serum IgE. Management strategies include allergen avoidance, pharmacotherapy (e.g. antihistamines, nasal corticosteroids, bronchodilators), and immunotherapy.^(9–12)^ Beyond genetic factors, this increase in allergic cases can be linked to various environmental and lifestyle changes. These include rising levels of air pollution, impacts of climate change, prolonged indoor stays, decreased physical activity, and alterations in dietary patterns.^(13,14)^ Nowadays, people’s eating habits are characterised by an increasing emphasis on convenience and speed, which frequently leads to greater intake of processed, altered, and modified foods; lower consumption of fruits and vegetables; and excessive amounts of sugar, saturated fat, and junk food.^(2,15)^ One of the healthiest diets is MD, which is based on traditional cuisines of countries bordering the Mediterranean Sea. It comprises whole grains, vegetables, beans, fruits, nuts, and seeds. Olive oil, the primary source of fat, is the most vital component of MD. The consumption of moderate amounts of fish, seafood, dairy products, and poultry is also a component of this dietary pattern. In contrast, red meat, sweets, sugary drinks, and butters are rarely consumed.^(2,14,16)^

MD offers a range of health benefits, including reducing the risk of CVD and metabolic syndrome, as well as supporting a healthy balance of gut microbiota.^(17)^ It helps individuals maintain healthy levels of glucose, blood pressure, and cholesterol. Recent research also indicates that MD may be beneficial for individuals with allergies. This could be because MD contains high levels of antioxidants (vitamins, minerals, and fatty acids) and anti-inflammatory substances, including polyphenols (e.g. oleuropein, hydroxytyrosol from olive oil, flavonoids from fruits and vegetables), omega-3 PUFA (EPA, DHA from fish), carotenoids, and dietary fibre, all of which have documented anti-inflammatory and immunomodulatory properties that promote immune function.^(2,18,19)^

While AD represent a significant burden in high-income countries, they are increasingly prevalent in low- and middle-income regions where urbanisation and industrialisation contribute to dietary transitions. These transitions often result in reduced consumption of traditional foods and greater intake of ultra-processed foods, paralleling the trends seen in Western dietary patterns. MD, historically rooted in countries bordering the Mediterranean Sea, offers a dietary model that could counteract these trends by emphasising nutrient-dense, anti-inflammatory foods such as fruits, vegetables, nuts, and olive oil. Importantly, the principles of MD can be adapted to diverse cultural and economic contexts, allowing global populations to benefit from its health-promoting effects even without direct access to specific Mediterranean food staples.^(6)^

However, existing research examining whether higher adherence to the MD is associated with a lower prevalence of AD is limited and inconsistent. Although some studies have suggested that MD adherence may reduce the likelihood or severity of certain allergic conditions, there remains a lack of comprehensive systematic reviews that synthesise these findings across different allergic outcomes. Therefore, this systematic review and meta-analysis aimed to determine the association between MD and allergies, including asthma, allergic rhinitis, atopic eczema, and FA. This review seeks to inform dietary strategies and public health recommendations that transcend regional boundaries by identifying the protective role of MD across various demographic and geographic populations. Emphasising the adaptability of MD to non-Mediterranean settings also underscores its potential to address public health challenges in diverse socio-economic contexts. These findings could have significant public health implications, potentially informing dietary recommendations and policy decisions aimed at reducing the burden of AD.

## Methods

The current review followed the PRISMA 2020 statement for systematic reviews^(20)^ (PRISMA Checklist). The review protocol was registered in the International Prospective Register of Systematic Reviews (CRD42023408724). No experiments were conducted with the human participants.

### Search strategy

A systematic literature search was conducted in PubMed, Scopus, ScienceDirect, and EBSCO from database inception to April 2024. The search strategy used combinations of the following terms with Boolean operators: (‘Mediterranean diet’ OR ‘Mediterranean dietary pattern’) AND (‘allergy’ OR ‘asthma’ OR ‘wheezing’ OR ‘rhinitis’ OR ‘eczema’ OR ‘food allergy’). Reference lists of relevant articles were also screened to identify additional eligible studies. No language restrictions were imposed. Search and study selection were performed by two independent reviewers (E.P. and S.A.N.). In cases where there were discrepancies between the two primary reviewers, a third reviewer was involved in resolving disagreements (E.A.).

### Eligibility criteria

All eligible studies were selected using the PICOS (Population, Intervention/Exposure, Comparison, Outcomes, Study design) framework (Table 1). Studies were included if they involved children (0–18 years), adults (>18 years), or pregnant women with offspring outcomes assessed, with or without a diagnosis of AD (asthma, wheezing, allergic rhinitis, atopic eczema, or FA). Eligible studies assessed adherence to the Mediterranean diet (MD) using a validated scoring instrument (e.g. KIDMED, Mediterranean diet score (MDS)) or a study-specific MD adherence index and compared higher versus lower adherence levels (or absence of a Mediterranean dietary pattern). Outcomes of interest included the prevalence, incidence, odds, or clinical severity/control of AD. Observational (cross-sectional, cohort, case–control) and interventional (randomised controlled) studies published between 2000 and April 2024 were eligible. Studies were excluded if they were conducted in animal models, did not assess adherence to a Mediterranean dietary pattern, or did not report relevant allergic outcomes. After the database search, duplicates were removed. Titles and abstracts were screened for eligibility according to predefined criteria, and full texts were retrieved and assessed when eligibility was unclear.

**Table 1.** PICOS criteria for inclusion of studies Table 1 long description.

Parameter	Description
**Population**	Children (0–18 years), adults (>18 years), and pregnant women (with offspring outcomes assessed), with or without a diagnosis of allergic disease (asthma, allergic rhinitis, atopic eczema, or food allergy)
**Intervention/Exposure**	Higher adherence to the Mediterranean diet, assessed by a validated scoring instrument (e.g. KIDMED, MedDiet Score, or study-specific MD adherence score)
**Comparison**	Lower adherence to the Mediterranean diet (lowest category or score) or no specific dietary pattern
**Outcome**	Prevalence, incidence, or odds of allergic disease (asthma, wheezing, allergic rhinitis, atopic eczema, food allergy); or measures of disease severity/control in diagnosed individuals
**Study**	Observational studies (cross-sectional, cohort, casecontrol) and interventional studies (randomised controlled trials) published from 2000 to April 2024

### Data extraction

The author, year, sample size, country, study design, MDS, and outcomes (impact of MD on allergy symptoms) were extracted for all studies. The effects size was determined using the OR and 95% CI and extracted from eligible studies.

### Quality assessment

The National Heart, Lung, and Blood Institute (NHLBI) tool was used to assess the validity of observational, cohort and cross-sectional studies included in this systematic review. The tool consists of 14 items divided into three categories: selection bias, measurement bias, and confounding. Each item was aimed at assessing a particular aspect of the study’s design, data collection, and analysis. For each item, a score of ‘yes,’ ‘no,’ or ‘cannot determine’ was assigned based on whether the study met the item’s criteria. The score is based on a scale from 0 to 14, with higher scores indicating higher-quality studies.^(21)^

### Data analysis

Meta-analyses were conducted separately for each allergic condition (asthma, wheezing – a respiratory symptom commonly assessed in epidemiological studies of allergic airway disease – rhinitis, and eczema) using random-effects models to account for anticipated between-study heterogeneity. For each study, OR and 95% CI were extracted and natural-log-transformed (log OR) prior to pooling. Pooled effect sizes were estimated using the DerSimonian-Laird random-effects model with inverse-variance weighting. When studies reported adjusted and unadjusted estimates, adjusted OR were preferentially extracted. Studies were classified as assessing either disease prevalence/incidence in general populations or disease severity/control in diagnosed individuals, as indicated in Table 3 (†). No moderator variables were included in the models given the small number of studies per condition, which precluded reliable meta-regression. Between-study heterogeneity was quantified using the Tau^(2)^ estimate and assessed using the *I* ^2^ statistic and Cochran’s *Q* test. *I* ^2^ values of 25%, 50%, and 75% were interpreted as indicating low, moderate, and high heterogeneity, respectively. Publication bias was evaluated visually through inspection of forest plots and statistically using Egger’s regression test. All statistical analyses were performed using the ‘meta’ and ‘metafor’ packages in *R*.

## Results

### Study selection

**Figure 1.**
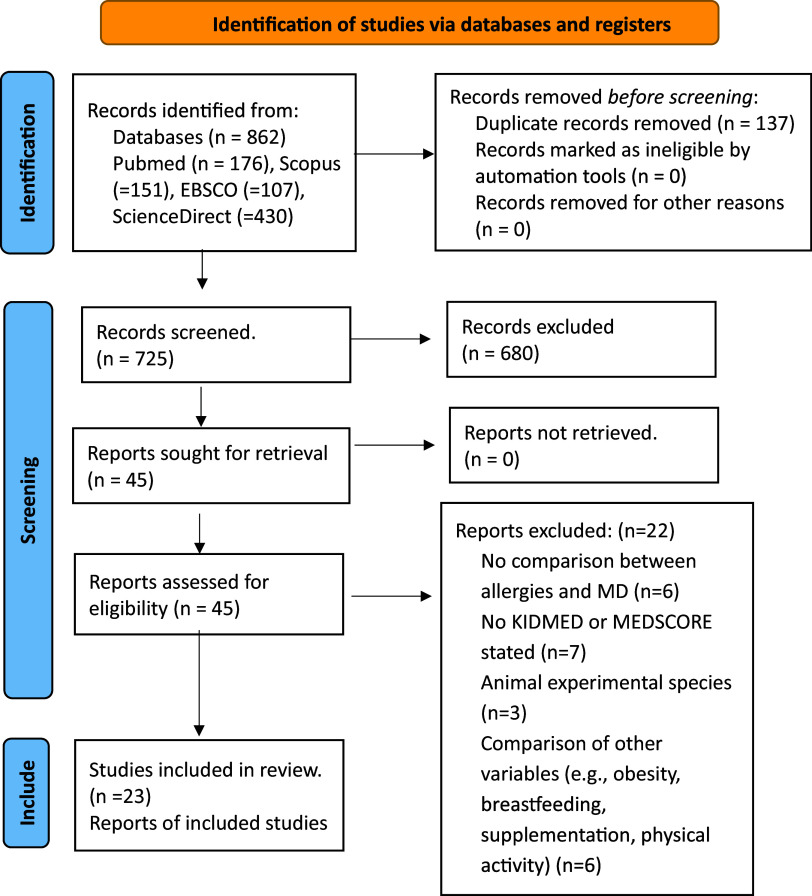
Schematic of selection of studies, PRISMA 2020. *Consider, if feasible to do so, reporting the number of records identified from each database or register searched (rather than the total number across all databases/registers). **If automation tools were used, indicate how many records were excluded by a human and how many were excluded by automation tools.

Of the 862 articles identified by the literature search, 137 were duplicates and 680 did not meet the inclusion criteria; therefore, they were excluded. Of the 45 articles evaluated for eligibility during the full-text screening, 22 did not meet the inclusion criteria. Finally, 23 articles met the inclusion criteria and were used for data extraction as shown in Figure 1.

### Risk of bias assessment

The NHLBI tool was used to evaluate the observational, cohort, and cross-sectional studies included in this systematic review (Table 2). Ten of the articles were rated ‘Good’ while thirteen were rated ‘fair’. A study that is considered fair means that it has good points and bad points, and that the results should be interpreted with caution. Fair-quality studies are generally free of major flaws; however, they may still have some limitations that could affect the validity and reliability of the findings. If a study is considered good, it means that it has few flaws and that the results are likely to be valid and reliable. Good-quality studies are typically well designed, employ appropriate methods for data collection and analysis, and are free of biases and confounding variables.

**Table 2.**
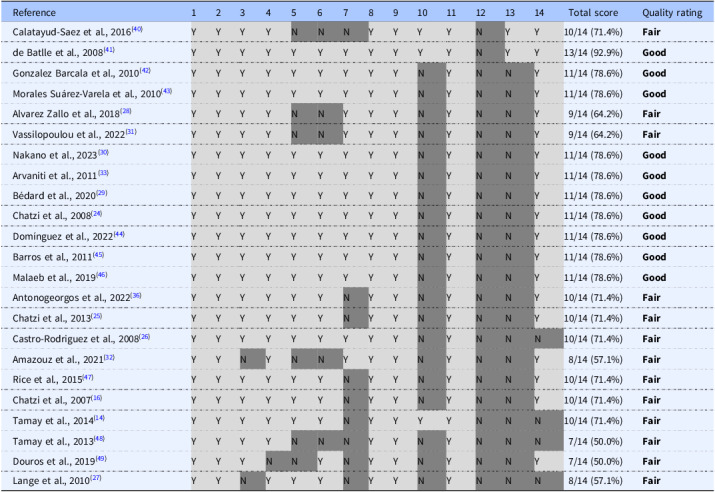
Risk of bias for observational cohort and cross-sectional studies Table 2 long description.

**Quality of included studies was assessed using the National Institutes of Health (NIH) Quality Assessment tool for Observational Cohort and Cross-Sectional Studies** (https://www.nhlbi.nih.gov/health-pro/guidelines/in-develop/cardiovascular-risk-reduction/tools/cohort). **1**. Was the research question or objective in this paper clearly stated? **2**. Was the study population clearly specified and defined? **3**. Was the participation rate of eligible persons at least 50%? **4**. Were all the subjects selected or recruited from the same or similar populations (including the same time)? Were inclusion and exclusion criteria for being in the study prespecified and applied uniformly to all participants? **5**. Was a sample size justification, power description, or variance and effect estimates provided? **6**. For the analyses in this paper, were the exposure(s) of interest measured prior to the outcome(s) being measured? **7**. Was the timeframe sufficient so that one could reasonably expect to see an association between exposure and outcome if it existed? **8**. For exposures that can vary in amount or level, did the study examine different levels of the exposure as related to the outcome (e.g. categories of exposure, or exposure measured as continuous variable)? **9**. Were the exposure measures (independent variables) clearly defined, valid, reliable, and implemented consistently across all study participants? **10**. Was the exposure(s) assessed more than once over time? **11**. Were the outcome measures (dependent variables) clearly defined, valid, reliable, and implemented consistently across all study participants? **12**. Were the outcome assessors blinded to the exposure status of participants? **13**. Was loss to follow-up after baseline 20% or less? **14**. Were key potential confounding variables measured and adjusted statistically for their impact on the relationship between exposure(s) and outcome(s)? **Total Score:** Number of yes; **NA,** not applicable; **N,** no; **Y,** yes. **Quality Rating:** Poor <50%, Fair 50–75%, Good ≥75%.

### Study characteristics

The current systematic review included a total of 23 studies, of which 15 were cross-sectional, 4 were cohort, 3 were population-based, and 1 was a case–control study. Eight studies were conducted in Spain, six in Greece, two in Turkey, one in the UK, one in France, one in the USA, one in Peru, one in Lebanon, one in Mexico, and one in Japan. The studies included 111,583 subjects of different ages (from children to adults). Table 3 provides a summary of the characteristics of the studies, including author, year, sample size, country, study design, MDS, allergy type, effect size, *p* value, and the effect of the MD on allergy symptoms.

**Table 3.** Summary of studies characteristics reporting any association between MD and allergies Table 3 long description.

R*	Country	Sample (*N*)	Age	Study design	KIDMED score	Med score	Score points range	Adherence to Med diet	Type of allergy	Effect size (OR)	95% Cl	*p*-Value	Beneficial effect	Conclusion(s)
^40^	Spain	104	1–5 yrs old	Observational	Yes	No	N/A	N/A	Asthma^†^	N/A	N/A	N/A	Yes	During the year of treatment, 32.2% of children had no bronchial hyperreactivity episodes, 35.3% had only one episode, and 24.9% had two episodes, compared to an average of 4.73 episodes in the previous year
^41^	Mexico	1476	6–7 yrs old & Mother	Cross-sectional study	No	Yes	0–8,0	3.7	Asthma	0.60	0.40–0.91	0.034	Yes	MD was found to be inversely related to asthma. (OR = 0.60, 95% CI = 0.40–0.91)
									Wheeze	0.64	0.47–0.87	0.001	Yes	MD was found to be inversely related to wheezing (0.64, 0.47–0.87)
									Rhinitis	0.41	0.22–0.77	0.046	Yes	MD was found to be inversely related to rhinitis (0.41, 0.22–0.77)
^42^	Spain	7354	6–7 yrs old	Cross-sectional study	No	Yes	0–22	13.1	Asthma	2.26	1.21–4.22	N/A	No	Higher asthma prevalence with higher adherence to MD {adjOR 2.26 (1.21–4.22)}
^43^	Spain	13,153	6–7 years old	Cross-sectional study	No	Yes	0–22,0	13.26	Eczema	1.03	0.99–1.08	0.071	No	No beneficial effect for Med Diet on atopic eczema (*P* = 0.071, OR = 1.03, 95% CI = 0.99–1.08)
^28^	Spain	1087 infants	Infants	Cross-sectional study	No	Yes	0–22	N/A	Wheeze	N/A	N/A	0.372	No	No beneficial effect: wheezing (*p* = 0.372)
									Eczema	N/A	N/A	0.315	No	No beneficial effect: wheezing (*p* = 0.315)
^31^	Greece	96	Mothers–infants	Observational	No	Yes	0–55	33.87	FPIAP**	N/A	N/A	0.023	Yes	MD appeared to protect against FPIAP {(olive oil *p* = 0.023, and during breastfeeding *p* = 0.003), vegetables (pregnancy,*p* = 0.008,and during breastfeeding *p* = 0.018)}
^30^	Japan	46,532	Mothers – child pairs	Cross-sectional study	No	Yes	0–9	4.05	Asthma	0.896	0.835–0.962	0.002	Yes	Beneficial differences were found in asthma (*p* = 0.002) - [0.896, 95% CI: 0.835, 0.962]
									Rhinitis	N/A	N/A	0.34	No	No significant difference in rhinitis (*p* = 0.340)
									Eczema	N/A	N/A	0.373	No	No significant difference were found atopic dermatitis (*p* = 0.373)
									Food allergy	N/A	N/A	*p* = 0.324	No	No significant differences in food allergy (*p* = 0.324)
^33^	Greece	700	10–12 years old	Cross-sectional study	Yes	No	0–12,0	4.8	Asthma	0.86	0.75–0.98	0.002	Yes	Greater adherence to the MD was inversely associated with any asthma symptoms (*p* < 0.005) [0.86 0.75–0.98]
^29^	UK	8629	Mother – child pairs	Cohort	No	Yes	0,0–7,0	3,9	Asthma	0.94	0.81–1.09	0.41	No	MD is not associated with a reduced risk of asthma {asthma: (*p* = 0.41) - [OR 0.94 (0.81–1.09) 95% CI]
									Wheeze	1.02	0.88–1.19	0.8	No	MD is not associated with a reduced risk of wheeze (*p* = 0.80) - [OR 1.02 (0.88–1.19)
									Eczema	0.07	1.13	0.99–1.29	No	MD is not associated with a reduced risk of eczema (*p* = 0.07)
^24^	Spain	460	6.5 years old	Cohort	No	Yes	0–12	N/A	Wheeze	0.22	0.08–0.58	0.002	Yes	Beneficial effect: wheeze (adjOR = 0.22 (0.08–0.58), atopic wheeze (adjOR = 0.30 (0.10–0.9), and atopy (adjOR = 0.55 (0.31–0.97)
^44^	Spain	2091	6–7 years old	Cross-sectional study	No	Yes	0–22 0–44	11.6 24.0	Asthma	1.1	0.9–1.2	0.336	No	Asthma (MDS22 *p* = 0.336)
^45^	Portugal	174	40 years old	Cross-sectional study	No	Yes	0–9	5	Asthma†	0.22	0.05–0.85	0.028	Yes	Adherence to MD had a 78% reduction in the risk of having asthma (OR = 0.22; 95% CI = 0.05–0.85; *P*-trend = 0.028)
^46^	Lebanon	1000	10.34 ± 3.96	Cross-sectional study	No	Yes	N/A	N/A	Asthma	0.23	0.090–0.956	0.002	Yes	Adherence to MD (occasional and daily consumption) was associated with lower odds with current asthma (*p* = 0.002)
^36^	Greece	1934	13–14 years old	Cross-sectional study	Yes	No	0–12,0	4.95	Asthma	<0.001	0.19–0.86	0.41	Yes	Adolescents with moderate and good MD adherence had 34 and 60% lower odds of asthma symptoms (*p* < 0.001)
									Rhinitis	<0.001	0.40–0.84	0.58	Yes	Adolescents with moderate and good MD adherence had 41% lower odds of allergic rhinitis symptoms, respectively (*p* < 0.001)
^25^	Greece & Spain	1771		Cohort	No	Yes	0–8	3.0	Wheeze	0.97	0.77–1.24	0.407	No	MD adherence was not associated with the risk of wheeze (*p* value = 0.407)
									Eczema	1.22	0.87–1.7	0.58	No	MD adherence was not associated with the risk of eczema (*p* value = 0.580)
^26^	Spain	1784	4.08 ± 0.8 years old	Cross-sectional	No	Yes	0–22	N/A	Wheeze	0.6	0.4–0.9	0.037	Yes	Adherence to MD had lower risk of wheezing (OR 0.6; 95% CI 0.4 to 0.9; *p* = 0.037).
^32^	France	975	8 years old	population-based study	Yes	No	0,0–8,0	3,32	Asthma	0.19	0.04–0.85	0.01	Yes	Beneficial effect of MD for asthma (KIDMED – *p* = 0.05 & MDS – *p* = 0.01)
^47^	Peru	287	9–19 years old	Case–control	No	Yes	0–22	N/A	Asthma	0.55	0.33,0.92	0.023	Yes	Decreased odds of asthma [OR = 0.55, 95 % CI (0.33,0.92), *p* = 0.02]
^16^	Greece	690	7–18 years old	Cross-sectional study	Yes	No	N/A	N/A	Rhinitis	0.34	0.18–0.64	<0.01	Yes	Beneficial effect *P* < 0,01
^14^	Turkey	9875	6–7 years old	Cross-sectional	No	Yes	0–22,0	N/A	Rhinitis	0.99	0.99–1.06	0.78	No	No beneficial effect with lifetime rhinitis (*p* = 0.78)
^48^	Turkey	9991	13–14 years old	Cross-sectional	No	Yes	0–22,0	12.35	Rhinitis	N/A	N/A	0.3	No	No beneficial effect with physician-diagnosed allergic rhinitis (*p* = 0.30)
^49^	Greece	44	5–15 years old	Cross-sectional study	Yes	No	0–12	5.7	Asthma†	N/A	N/A	0.007	Unclear	The paper examines three cytokines and demonstrates that there is a correlation, but it varies. So, the results are inconclusive.
^27^	USA	1376	Mother–infant pairs	Cohort	No	Yes	0–9	4.6	Wheeze	0.92	0.77–1.08	0.06	No	No beneficial effect of maternal dietary pattern on wheeze (OR, 0.92 [0.77–1.08])

*R: Reference, **FPIAP: food protein-induced allergic proctocolitis, ^†^Severity/control study: outcome was assessed in participants with an established diagnosis of the allergic condition (disease severity, symptom control, or inflammatory markers). All unmarked studies assessed prevalence or incidence in general or population-based samples.

Eleven studies met the inclusion criteria for the meta-analysis of MD and asthma, encompassing a combined sample size of 71,152 participants. The studies varied in design, including 8 cross-sectional studies, 1 cohort study, 1 case–control study and 1 observational study. The studies were conducted across various countries, including Spain, Mexico, Greece, Japan, the UK, Lebanon, Turkey, Portugal, France, Peru, and the USA, providing a diverse sample population. MD adherence was assessed using various scoring systems, with some studies utilising the KIDMED test and others creating their own Med Score based on dietary components.

Six studies met the inclusion criteria for the meta-analysis of MD and wheeze, encompassing a combined sample size of 15,496 participants. The studies varied in design, including two cross-sectional and four cohort studies. The studies were conducted across various countries, including Mexico, Spain, the UK, Greece, and the USA, providing a diverse sample population. MD adherence was assessed using their own Med scores based on dietary components.

Four studies met the inclusion criteria for the meta-analysis of MD and rhinitis and encompass a combined sample size of 13,975 participants. All studies were cross-sectional. The studies were conducted across various countries, including Mexico, Greece, and Turkey, providing a diverse sample population. MD adherence was assessed using various scoring systems, with some studies utilising the KIDMED test and others creating their own Med Score based on dietary components.

Three studies met the inclusion criteria for the meta-analysis of MD and eczema, encompassing a combined sample size of 23,553 participants. The studies varied in design, including one cross-sectional and two cohort studies. The studies were conducted in the UK, Spain and Greece. MD adherence was assessed using the Med Score with one study creating its own scoring system based on dietary components.

### Meta-analysis of MD and asthma

**Figure 2.**
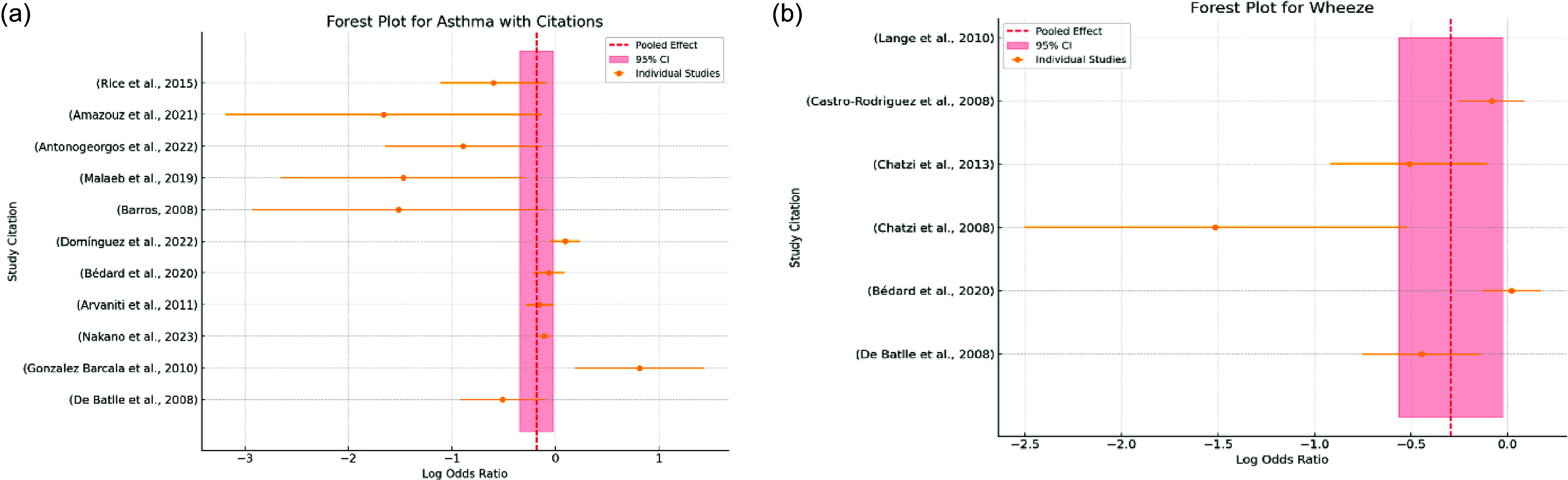
Forest plots showing the effects of the MD on (a) asthma and (b) wheeze.

The meta-analysis for asthma included 11 qualified studies, with a pooled random-effects mean log OR of −0.18 (95% CI: −0.34, −0.02). This indicated a statistically significant protective effect of adherence to MD on asthma risk. Most individual studies reported an OR below 1, reflecting consistent findings of reduced asthma incidence among those adhering to the MD. Notably, studies by Nakano et al. (2023) and Barros (2008) showed strong protective effects, whereas others, such as Gonzalez Barcala et al. (2010), reported weaker or non-significant associations. Moderate heterogeneity was observed (Tau^2^ = 0.03), suggesting some variability in the study populations, methodologies, or adherence measures used. These findings support the potential role of MD in reducing asthma risk, although this variability emphasises the need for further investigation (Table 4 and Figure 2a).

**Table 4.** Summary of studies included in the meta-analysis for asthma Table 4 long description.

R^*^	Country	Sample (*N*)	Age	Study design	KIDMED score	Med score	Score points range	Adherence to Med diet	Type of allergy	Effect size (OR)	95% Cl	*p*-Value	Beneficial effect	Conclusion(s)
^40^	Spain	104	1–5 yrs old	Observational	Yes	No	N/A	N/A	Asthma	N/A	N/A	N/A	Yes	During the year of treatment, 32.2% of children had no bronchial hyperreactivity episodes, 35.3% had only one episode, and 24.9% had two episodes, compared to an average of 4.73 episodes in the previous year
^41^	Mexico	1476	6–7 yrs old & Mother	Cross-sectional study	No	Yes	0–8,0	3.7	Asthma	0.60	0.40–0.91	0.034	Yes	MD was found to be inversely related to asthma. (OR = 0.60, 95% CI = 0.40–0.91)
^42^	Spain	7354	6–7 yrs old	Cross-sectional study	No	Yes	0–22	13.1	Asthma	2.26	1.21–4.22	N/A	No	Higher asthma prevalence with higher adherence to MD {adjOR 2.26 (1.21–4.22)}
^30^	Japan	46,532	Mothers – child pairs	Cross-sectional study	No	Yes	0–9	4.05	Asthma	0.896	0.835–0.962	0.002	Yes	Beneficial differences were found in asthma (*p* = 0.002) - [0.896, 95% CI: 0.835, 0.962]
^33^	Greece	700	10–12 years old	Cross-sectional study	Yes	No	0–12,0	4.8	Asthma	0.86	0.75–0.98	0.002	Yes	Greater adherence to the MD was inversely associated with any asthma symptoms (*p* < 0.005) [0.86 0.75–0.98]
^29^	UK	8629	Mother- child pairs	Cohort	No	Yes	0,0–7,0	3,9	Asthma	0.94	0.81–1.09	0.41	No	MD is not associated with a reduced risk of asthma {asthma: (*p* = 0.41) - [OR 0.94 (0.81–1.09) 95% CI]
^44^	Spain	2091	6–7 years old	Cross-sectional study	No	Yes	0–22 0–44	11.6 24.0	Asthma	1.1	0.9–1.2	0.336	No	Asthma (MDS22 *p* = 0.336)
^45^	Portugal	174	40 years old	Cross-sectional study	No	Yes	0–9	5	Asthma	0.22	0.05–0.85	0.028	Yes	Adherence to MD had a 78% reduction in the risk of having asthma (OR = 0.22; 95% CI = 0.05–0.85; *P*-trend = 0.028)
^46^	Lebanon	1000	10.34 ± 3.96	Cross-sectional study	No	Yes	N/A	N/A	Asthma	0.23	0.090–0.956	0.002	Yes	Adherence to MD (occasional and daily consumption) was associated with lower odds with current asthma (*p* = 0.002)
^36^	Greece	1934	13–14 years old	Cross-sectional study	Yes	No	0–12,0	4.95	Asthma	<0.001	0.19–0.86	0.41	Yes	Adolescents with moderate and good MD adherence had 34 and 60% lower odds of asthma symptoms (*p* < 0.001)
^32^	France	975	8 years old	Population-based study	Yes	No	0,0–8,0	3,32	Asthma	0.19	0.04–0.85	0.01	Yes	Beneficial effect of MD for asthma (KIDMED – *p* = 0.05 & MDS – *p* = 0.01)
^47^	Peru	287	9–19 years old	Case–control	No	Yes	0–22	N/A	Asthma	0.55	0.33,0.92	0.023	Yes	Decreased odds of asthma [OR = 0.55, 95 % CI (0.33,0.92), *p* = 0.02]
^49^	Greece	44	5–15 years old	Cross-sectional study	Yes	No	0–12	5.7	Asthma	N/A	N/A	0.007	Unclear	The paper examines three cytokines and demonstrates that there is a correlation, but it varies. So, the results are inconclusive.

* R: Reference.

### Meta-analysis of MD and wheeze

The meta-analysis for wheeze included six studies, with a pooled random-effects log OR of −0.29 (95% CI: −0.56, −0.03). This indicated a statistically significant protective effect of adherence to MD on the risk of wheezing. Among the individual studies, most demonstrated a beneficial effect, with OR below 1, suggesting a reduction in wheeze risk. For example, Chatzi et al. (2008) reported a strong protective effect with an OR of 0.22 (95% CI: 0.08–0.58, *p* = 0.002), while Castro-Rodriguez et al. (2008) showed an OR of 0.6 (95% CI: 0.4–0.9, *p* = 0.037). However, some studies, such as Lange et al. (2010), reported no significant association, with an OR of 0.92 (95% CI: 0.77–1.08, *p* = 0.06). Moderate heterogeneity was observed (Tau^2^ = 0.06), reflecting differences in study methodologies, populations, or MD adherence definitions (Table 5 and Figure 2b).

**Table 5.** Summary of studies included in the meta-analysis for wheeze Table 5 long description.

R^*^	Country	Sample (*N*)	Age	Study design	KIDMED score	Med score	Score points range	Adherence to Med diet	Type of allergy	Effect size (OR)	95% Cl	*p*-Value	Beneficial effect	Conclusion(s)
^41^	Mexico	1476	6–7 yrs old & Mother	Cross-sectional study	No	Yes	0–8,0	3.7	Wheeze	0.64	0.47–0.87	0.001	Yes	MD was found to be inversely related to wheezing (0.64, 0.47–0.87)
^28^	Spain	1087 infants	Infants	Cross-sectional study	No	Yes	0–22	N/A	Wheeze	N/A	N/A	0.372	No	No beneficial effect: wheezing (*p* = 0.372)
^29^	UK	8629	Mother- child pairs	Cohort	No	Yes	0,0–7,0	3,9	Wheeze	1.02	0.88–1.19	0.8	No	MD is not associated with a reduced risk of wheeze (*p* = 0.80) - [OR 1.02 (0.88–1.19)
^24^	Spain	460	6.5 years old	Cohort	No	Yes	0–12	N/A	Wheeze	0.22	0.08–0.58	0.002	Yes	Beneficial effect: wheeze (adjOR = 0.22 (0.08–0.58), atopic wheeze (adjOR = 0.30 (0.10–0.9), and atopy (adjOR = 0.55 (0.31–0.97)
^25^	Greece & Spain	1771		Cohort	No	Yes	0–8	3.0	Wheeze	0.97	0.77–1.24	0.407	No	MD adherence was not associated with the risk of wheeze (*p* value = 0.407)
^26^	Spain	1784	4.08 ± 0.8 years old	Cross-sectional	No	Yes	0–22	N/A	Wheeze	0.6	0.4–0.9	0.037	Yes	Adherence to MD had lower risk of wheezing (OR 0.6; 95% CI 0.4 to 0.9; *p* = 0.037).
^27^	USA	1376	Mother–infant pairs	Cohort	No	Yes	0–9	4.6	Wheeze	0.92	0.77–1.08	0.06	No	No beneficial effect of maternal dietary pattern on wheeze (OR, 0.92 [0.77–1.08])

* R: Reference.

### Meta-analysis of MD and rhinitis

**Figure 3.**
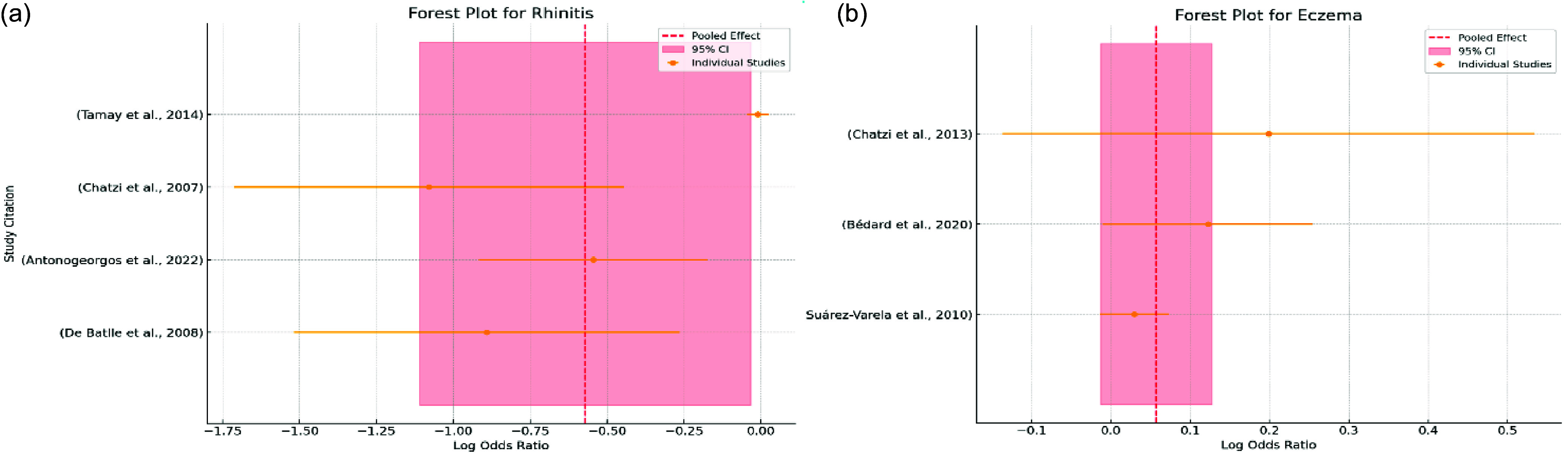
Figure 3 long description. Forest plots showing the effects of the MD on (a) rhinitis and (b) eczema.

The meta-analysis for rhinitis included four studies, with a pooled random-effects log OR of −0.54 (95% CI: −1.00, −0.08). This result indicates a statistically significant protective effect of adherence to the MD on the risk of rhinitis. The effect sizes varied among the individual studies. De Batlle et al. (2008) reported an OR of 0.41 (95% CI: 0.22–0.77, *p* = 0.046), demonstrating a strong protective effect. Similarly, Antonogeorgos et al. (2022) and Chatzi et al. (2007) found beneficial associations, with ORs of 0.58 (95% CI: 0.40–0.84, *p* < 0.001) and 0.34 (95% CI: 0.18–0.64, *p* < 0.01), respectively. In contrast, Tamay et al. (2014) found no significant association, reporting an OR of 0.99 (95% CI: 0.99–1.06, *p* = 0.78). Moderate heterogeneity was observed (Tau^2^ = 0.04), suggesting some variability across studies (Table S1 and Figure 3a).

### Meta-analysis of MD and eczema

The meta-analysis for eczema included three studies, with a pooled random-effects log OR of 0.09 (95% CI: −0.02, 0.20), indicating no statistically significant association between adherence to MD and eczema risk. Among the included studies, Morales Suárez-Varela et al. (2010) reported an OR of 1.03 (95% CI: 0.99–1.08, *p* = 0.071), suggesting no beneficial effect of MD on atopic eczema. Similarly, Bédard et al. (2020) found no association, with an OR of 1.13 (95% CI: 0.99–1.29, *p* = 0.07). Chatzi et al. (2013) also observed no significant protective effect, with an OR of 1.22 (95% CI: 0.87–1.7, *p* = 0.58). These findings suggest that adherence to the MD does not significantly reduce the risk of eczema. Moderate heterogeneity (Tau^2^ = 0.03) was observed, reflecting some variability across studies (Table S2 and Figure 3b).

### Qualitative synthesis of MD and FA

Two studies were identified and analysed. A study by Vassilopoulou et al. (2022) indicated a beneficial effect of MD on food protein-induced allergic proctocolitis (FPIAP). The study found that adherence to MD, particularly the consumption of olive oil and vegetables during pregnancy and breastfeeding, was associated with a reduced risk of FPIAP (*p* = 0.023). In contrast, the study by Nakano et al. (2023) did not find a significant association between adherence to the MD and the incidence of FA in mothers and child pairs (*p* = 0.324). Due to the lack of quantitative data (ORs and CIs), a formal heterogeneity assessment could not be performed. Given the small number of included studies, conclusions regarding the sources of observed heterogeneity are limited, and these results should be interpreted with caution. A formal assessment of publication bias was not possible due to the limited number of studies and the lack of quantitative data. However, the inclusion of studies with both beneficial and non-beneficial findings help mitigate concerns about publication bias.

## Discussion

This systematic review and meta-analysis aimed to assess the impact of MD on allergic conditions, including asthma, allergic rhinitis, atopic eczema, and FA. These findings highlight the potential protective effects of MD, particularly against asthma and respiratory allergies.

The results of this meta-analysis indicate that adherence to MD is associated with a statistically significant reduction in the risk of asthma, as evidenced by the pooled log OR of −0.18 (95% CI: −0.34, −0.02). This finding aligns with the hypothesis that anti-inflammatory and antioxidant properties of MD, derived from its high content in fruits, vegetables, and omega-3 fatty acids, may positively influence respiratory health.^(2,15)^ These components likely mitigate airway inflammation, which is a key mechanism in asthma pathogenesis.

The observed heterogeneity (Tau^2^ = 0.03) may reflect differences in dietary adherence scoring systems (e.g. KIDMED, MedDiet Score), population demographics (e.g. children vs. adults), and study designs (e.g. cross-sectional vs. cohort). For instance, studies conducted in Mediterranean countries, such as those by Barros (2008) and Arvaniti et al. (2011), tended to report stronger protective effects, potentially due to higher baseline adherence to the MD compared to studies from non-Mediterranean regions. It should also be noted that many Mediterranean-region studies were conducted exclusively in paediatric populations, where immune system maturation, dietary habits, and disease phenotypes differ from those of adults. The stronger associations observed in these studies may therefore reflect age-related differences in susceptibility to the anti-inflammatory and antioxidant properties of the MD, rather than geographic or cultural factors alone. Because the pooled estimate for asthma combined studies of children, adolescents, adults, and prenatal/maternal exposures, the overall effect size may obscure differential responses across age groups. This reinforces the need to study how culturally appropriate adaptations of the MD could benefit populations in non-Mediterranean regions, where traditional diets may lack key MD components such as olive oil or fresh seafood. Future studies should stratify analyses by age group and explore affordable and locally available alternatives to ensure their widespread applicability.

Despite the significant pooled effect, some individual studies, such as Gonzalez Barcala et al. (2010), did not observe a protective association, which may be due to methodological differences or unmeasured confounders such as socio-economic status, physical activity, or environmental exposures. The findings underscore the importance of standardised MD adherence definitions and comprehensive adjustment for confounders in future research. While the overall evidence supports the use of MD as a potential dietary strategy for asthma prevention, further longitudinal studies are needed to confirm the causal relationships and explore the mechanisms involved, particularly across diverse populations and regions.

The analysis also revealed a significant protective effect of the MD against wheeze, respiratory symptom commonly assessed in epidemiological studies of allergic airway disease, with a pooled log OR of −0.29 (95% CI: −0.56, −0.03). These results are consistent with the anti-inflammatory and antioxidant properties of MD, which reduce airway inflammation and promote respiratory health. Key components, such as fruits, vegetables, olive oil, and omega-3 fatty acids, likely contribute to these benefits. Stronger protective effects have been observed in Mediterranean populations (e.g. Chatzi et al., 2008; Castro-Rodriguez et al., 2008), where adherence levels tend to be higher. Conversely, studies conducted in non-Mediterranean regions, such as Lange et al. (2010), found no significant associations, potentially because of cultural and dietary differences or unmeasured factors. This highlights the need for educational initiatives and public health strategies to promote MD adherence in non-Mediterranean regions, particularly in populations where Western dietary patterns are prevalent. Such programmes could focus on affordable substitutes and foster dietary transitions towards higher intakes of plant-based foods and healthy fats.

The moderate heterogeneity observed (Tau^2^ = 0.06) highlights the need for caution in interpreting these findings. Variations in the study design (e.g. cohort vs. cross-sectional studies), population demographics (e.g. children vs. adults), and MD adherence scoring systems could account for these discrepancies. Furthermore, unmeasured factors such as environmental exposures, physical activity, or genetic predispositions may have influenced the results. Future research should focus on standardising MD adherence metrics, studying diverse populations, and investigating the mechanisms driving observed benefits. It is important to note that wheezing, unlike asthma, rhinitis, or eczema, is a clinical symptom rather than a distinct disease. In the epidemiological literature, wheezing is frequently used as an indicator of airway hyper-responsiveness, particularly in young children where formal asthma diagnosis is often delayed due to the variable natural history of early respiratory symptoms.^(22,23)^ Several of the studies included in the present study assessed parent-reported wheezing episodes in preschool or school-age populations.^(24–29)^ A proportion of affected children may go on to develop asthma while others may experience transient symptoms that resolve with age. This distinction has implications for the interpretation of our pooled estimate. Thus, the protective association observed between MD adherence and wheezing (log OR: −0.29; 95% CI: −0.56, −0.03) may capture a broad spectrum of airway phenotypes, ranging from transient viral-induced wheeze to early-onset atopic asthma, each of which may respond differently to dietary exposures. Future studies would benefit from distinguishing between wheezing phenotypes (e.g. transient early wheeze, persistent wheeze, and late-onset wheeze) and from reporting physician-confirmed asthma diagnoses alongside symptom-based outcomes, as this would allow for more precise estimation of the role of MD in allergic airway disease. For rhinitis, adherence to the MD was associated with a significant protective effect, with a pooled log OR of −0.54 (95% CI: −1.00, −0.08). These results align with the existing evidence supporting the anti-inflammatory and antioxidant properties of MD. High intake of fruits, vegetables, olive oil, and omega-3 fatty acids in the MD may contribute to reduced airway inflammation, thereby mitigating the risk of rhinitis.

The individual study findings showed some variability. The strongest protective effects were reported by Chatzi et al. (2007) and De Batlle et al. (2008), both of which were conducted in Mediterranean or high-adherence populations. These results underscore the potential for stronger benefits of MD in regions with higher adherence. Still, achieving similar benefits in populations with lower adherence levels will require tailored dietary interventions that consider cultural preferences and logistical barriers, such as availability and affordability of MD components in low- and middle-income regions. This underscores the importance of global dietary flexibility when translating the findings into practical recommendations. However, a study by Tamay et al. (2014), conducted in Turkey, found no significant association, which may reflect differences in dietary patterns, cultural factors, or unmeasured confounders.

Moderate heterogeneity (Tau^2^ = 0.04) was observed, likely due to differences in the study design (e.g. cross-sectional studies), population characteristics (e.g. age groups), and methods for assessing MD adherence. Furthermore, the limited number of studies and absence of longitudinal designs constrain the generalizability of these findings. Future research should include more diverse populations and utilise standardised MD adherence tools to improve comparability across studies.

In contrast, adherence to MD showed no significant protective effect against eczema, as evidenced by a pooled OR of 0.09 (95% CI: −0.02, 0.20). This finding was consistent across the included studies (e.g. Morales Suárez-Varela et al., 2010; Bédard et al., 2020; Chatzi et al., 2013), suggesting that the eczema’s complex pathogenesis may not be directly influenced by MD. Variability in study designs, populations, and MD adherence assessments likely contributed to moderate heterogeneity (Tau^2^ = 0.03). Future studies should examine the specific dietary components and their interactions with other risk factors to clarify the role of MD in eczema prevention.

This review found only two studies that investigated the association between MD and FA.^(30,31)^ Due to insufficient quantitative data (e.g. ORs, CIs), a meta-analysis for FA and FPIAP could not be performed. Although Vassilopoulou et al. (2022) reported a protective effect of MD components such as olive oil during breastfeeding, Nakano et al. (2023) found no significant associations. Notably, a recent systematic review exploring the effect of the dietary components of MD on FAs indicated an overall beneficial effect between the food components of MD and FA.^(5)^ The lack of standardised effect measures, combined with regional differences in dietary patterns, highlights the need for globally representative data that investigates these associations. Future research should focus on harmonising methodologies to capture the context-specific effects of diet on global FA.

Notably, previous research supports the MD’s potential in allergy prevention.^(15,32–34)^ Antonogeorgos (2024) demonstrated that higher MD adherence or increased fruit and vegetable intake reduced childhood asthma risk.^(34)^ A 2019 systematic review suggested that MD adherence during pregnancy could lower the risk of AD in offspring.^(35)^ Similarly, Garcia-Marcos et al. (2013) found that MD adherence was more protective against childhood asthma in Mediterranean populations than in non-Mediterranean populations.^(15)^ These effects may be attributed to the MD’s rich content of antioxidants, flavonoids, and polyphenols, which reduce inflammation and bolster immune responses.^(32,33,36)^ These findings reinforce the global applicability of MD adherence as a public health priority, not only within Mediterranean populations but also in regions experiencing dietary transitions towards processed foods. Public health campaigns that integrate MD principles into existing dietary traditions can amplify their global impacts.

Although the MD originates from countries bordering the Mediterranean Sea, its core principles, including high consumption of plant-based foods, legumes, whole grains, fruits, vegetables, and unsaturated fats, alongside reduced intake of ultra-processed foods - are adaptable to diverse cultural and socio-economic contexts through locally available and culturally appropriate food alternatives.^(37)^ Economic constraints, limited food access, and established regional dietary traditions may, however, impede adherence, particularly in low- and middle-income settings.^(38,39)^ It should also be noted that most studies informing this review were conducted in Mediterranean and high-income countries, which may limit the generalisability of findings to populations with differing food environments and resource availability.^(34)^ Consequently, public health strategies promoting Mediterranean-style dietary patterns should prioritise cultural acceptability, affordability, and context-specific adaptation; further research in underrepresented geographic and economic settings is thus warranted.

This study had several limitations. Variability across studies, including differences in MD adherence definitions, scoring systems (e.g. KIDMED and MedDiet Score), and population demographics, contributed to heterogeneity in the results. Formal sub-group analyses stratified by age would be valuable but were not feasible in the current study because the adult stratum contained only one study and the maternal/prenatal stratum contained only two to three studies per outcome, which is insufficient to produce reliable pooled estimates. Similarly, the included studies differed in outcome type and most reported disease prevalence or incidence, whereas a small number assessed severity or control in diagnosed individuals. Pooling these outcome types may introduce additional clinical heterogeneity, and future meta-analyses should consider pre-specified sub-group analyses by outcome type. In addition, MD adherence was assessed using different scoring systems across studies, which may limit comparability. These factors emphasise the need for standardised methodologies for future research. Potential biases, such as self-reported dietary intake and confounding variables (e.g. socio-economic status and physical activity), may also impact the findings.

## Conclusion

In conclusion, this meta-analysis provides evidence that adherence to the MD is associated with a significantly lower prevalence of asthma, wheezing, and rhinitis. These protective associations are biologically plausible given the shared role of airway inflammation in all three conditions and the established anti-inflammatory and antioxidant properties of key MD components, including fruits, vegetables, olive oil, and omega-3 fatty acids. No significant protective effect was found against eczema, suggesting that its predominantly barrier-driven pathogenesis may not be as readily influenced by dietary patterns. Further, quantitative data on FA precluded meta-analysis for that outcome, and further research with standardised effect measures is needed.

While the results support MD as a promising dietary approach for respiratory allergy prevention, variability across studies, such as differences in adherence definitions and study designs, underscores the need for standardised methodologies in future research. Stronger effects observed in Mediterranean-region and paediatric populations highlight the importance of age-stratified analyses and of investigating how MD principles can be adapted to non-Mediterranean dietary contexts. Future longitudinal studies should clarify causal pathways, prioritise diverse populations, and evaluate the feasibility and effectiveness of MD-based interventions for asthma, wheezing, and rhinitis prevention across different geographic and socio-economic settings.

## Table 1. Long description

A table with five rows and two columns. The columns are labeled Parameter and Description. Row 1: Parameter, Population; Description, Children (018 years), adults (18 years), and pregnant women (with offspring outcomes assessed), with or without a diagnosis of allergic disease (asthma, allergic rhinitis, atopic eczema, or food allergy). Row 2: Parameter, Intervention/Exposure; Description, Higher adherence to the Mediterranean diet, assessed by a validated scoring instrument (e.g. KIDMED, MedDiet Score, or study-specific MD adherence score). Row 3: Parameter, Comparison; Description, Lower adherence to the Mediterranean diet (lowest category or score) or no specific dietary pattern. Row 4: Parameter, Outcome; Description, Prevalence, incidence, or odds of allergic disease (asthma, wheezing, allergic rhinitis, atopic eczema, food allergy); or measures of disease severity/control in diagnosed individuals. Row 5: Parameter, Study; Description, Observational studies (cross-sectional, cohort, casecontrol) and interventional studies (randomised controlled trials) published from 2000 to April 2024.

Navigate back to Table 1..

## Table 2. Long description

The table evaluates the risk of bias for observational cohort and cross-sectional studies, rating them as Good or Fair. It includes 23 studies with columns for reference, 14 criteria (1 to 14), total score, and quality rating. Each row lists a study, its compliance with each criterion (Y for Yes, N for No), total score out of 14, and quality rating. Ten studies are rated Good, and thirteen are rated Fair. The table provides detailed information on the quality of each study based on specific criteria.

Navigate back to Table 2..

## Table 3. Long description

The table presents a summary of studies examining the association between the Mediterranean diet (MD) and various allergic conditions. It includes 26 rows and 14 columns. The columns are labeled as follows: Reference, Country, Sample size, Age, Study design, KIDMED score, Med score, Score points range, Adherence to Med diet, Type of allergy, Effect size (OR), 95% CI, p Value, Beneficial effect, and Conclusion. Each row provides details for a specific study, including the country where the study was conducted, the sample size, the age group of participants, the study design, whether a KIDMED score or Med score was used, the range of score points, adherence to the Mediterranean diet, the type of allergy investigated (asthma, wheezing, rhinitis, or eczema), the effect size (OR), the 95% confidence interval (CI), the p-value, whether a beneficial effect was observed, and the conclusion of the study. The table captures various studies with different sample sizes, age groups, and study designs, providing a comprehensive overview of the research on the relationship between the Mediterranean diet and allergic conditions.

Navigate back to Table 3..

## Table 4. Long description

The table presents a summary of studies examining the relationship between adherence to the Mediterranean diet and asthma risk. It includes 11 rows and 13 columns. The columns are labeled as follows: R*, Country, Sample (M), Age, Study design**, KIDMED score, Med score, Score points range, Adherence to Med diet, Type of allergy, Effect size (OR), 95% CI, p-Value, Beneficial effect, and Conclusion(s). Each row provides data for a specific study, detailing the country, sample size, age group, study design, KIDMED score, Mediterranean diet score, score points range, adherence to the Mediterranean diet, type of allergy, effect size (OR), 95% confidence interval, p-value, beneficial effect, and conclusions. The studies vary in sample size, age groups, and study designs, with some showing significant protective effects of the Mediterranean diet on asthma risk while others report weaker or non-significant associations.

Navigate back to Table 4..

## Table 5. Long description

The table summarizes studies included in the meta-analysis for wheeze, detailing various countries, sample sizes, ages, study designs, and outcomes. It has 6 rows and 13 columns. The columns are labeled as follows: R, Country, Sample (N), Age, Study design, KIDMED score, Med score, Score points range, Adherence to Med diet, Type of allergy, Effect size (OR), 95% CI, p-Value, Beneficial effect, and Conclusion(s). The rows provide data for each study, including the country, sample size, age group, study design, whether KIDMED score was used, the median score, score points range, adherence to Mediterranean diet, type of allergy, effect size with 95% confidence interval, p-value, whether a beneficial effect was observed, and the conclusions of the study.

Navigate back to Table 5..

## Figure 3. Long description

Panel A: Forest Plot for Rhinitis. The forest plot shows the effects of the Mediterranean diet on rhinitis. The x-axis represents the Log Odds Ratio, ranging from -1.75 to 0.00. The y-axis lists study citations: Tamay et al. 2014, Chatzi et al. 2007, Antonogeorgos et al. 2022, and De Batlle et al. 2008. Each study is represented by an orange dot with horizontal lines indicating the 95% confidence interval. A red dashed vertical line represents the pooled effect. Panel B: Forest Plot for Eczema. The forest plot shows the effects of the Mediterranean diet on eczema. The x-axis represents the Log Odds Ratio, ranging from -0.1 to 0.5. The y-axis lists study citations: Chatzi et al. 2013, Bedard et al. 2020, and Suarez-Varela et al. 2010. Each study is represented by an orange dot with horizontal lines indicating the 95% confidence interval. A red dashed vertical line represents the pooled effect.

Navigate back to Figure 3..
